# Study of Single Nucleotide Polymorphisms Associated with Breast Cancer Patients among Arab Ancestries

**DOI:** 10.1155/2022/2442109

**Published:** 2022-10-11

**Authors:** Yasser Osman, Tarek Elsharkawy, Tariq Mohammad Hashim, Jumana Abdulwahab Alratroot, Fatima Aljindan, Liqa Almulla, Hind Saleh Alsuwat, Waad Mohammed Al Otaibi, Fatma Mohammed Hegazi, Abdallah M. Ibrahim, J. Francis Borgio, Sayed AbdulAzeez

**Affiliations:** ^1^Pathology Department, College of Medicine, Imam Abdulrahman Bin Faisal University, Dammam 31441, Saudi Arabia; ^2^Department of Genetic Research, Institute for Research and Medical Consultations (IRMC), Imam Abdulrahman Bin Faisal University, Dammam 31441, Saudi Arabia; ^3^Department of Fundamentals of Nursing, College of Nursing, Imam Abdulrahman Bin Faisal University, Dammam 31441, Saudi Arabia

## Abstract

The aim of this study is to investigate the single nucleotide polymorphisms (SNPs) associated with breast cancer in our population of Arab patients. We investigated 26 breast cancer patients and an equal number of healthy age- and sex-matched control volunteers. We examined the exome wide microarray-based biomarkers and screened 243,345 SNPs for their possible significant association with our breast cancer patients. Successfully, we identified the most significant (*p* value ≤9.14 × 10^−09^) four associated SNPs [*SNRK* and *SNRK-AS1-*rs202018563G; *BRCA2-*rs2227943C; *ZNF484-*rs199826847C; and *DCPS-*rs1695739G] among persons with breast cancer versus the healthy controls even after Bonferroni corrections (*p* value <2.05 × 10^−07^). Although our patients' numbers were limited, the identified SNPs might shed some light on certain breast cancer-associated functional multigenic variations in Arab patients. We assert on the importance of more extensive large-scale analysis to confirm the candidate biomarkers and possible target genes of breast cancer among Arab ancestries.

## 1. Introduction

Breast cancer, as a multifactorial disease, is the most common cancer in the world [1]. The major risk factors associated with breast cancer appear to be environmental and genetic factors [2, 3]. Previous studies indicate that genetic factors account for about 27% of the breast cancer risk [4]. A few genes including *BRCA1*, *BRCA2*, and *ATM* have been known to be associated with the risk of breast cancer [5].

Yang et al. reported meta-analyses, including 14306 cases and 15099 controls group numbers from 13 case-control studies, and explored the association between the rs3803662 polymorphism and the risk of breast cancer. Their results indicated that rs3803662 is significantly associated with breast cancer risk in Caucasian women but did not find this association in Asian women [6].

In addition, Garcia et al. reported the association of XRCC4 c.1394G > T with breast cancer development among selected Filipinos [7]. The results by Garcia et al. supported the hypothesis that polymorphisms in the XRCC4 c.1394G > T gene may influence the functioning of the DNA repair pathway [7].

Node-like receptors (NLR) are a group of intracellular proteins that can detect microbes and abnormal signals. Thus, it could control various immune pathways. There are around 22 NLR proteins that has not been well studied [8]. *NLRC5* is one of the NLR proteins which is expressed mostly in the lymphoid and myeloid cells. The expression of *NLRC5* is found to have been induced strongly by INF-y [9]. Overexpressed *NLRC5* can repress the signal of NF-*κ*B– and AP-1–. Thus, in the absence of *NLRC5* expression, there will be an increased proinflammatory response. Therefore, *NLRC5* has a negative modulation effect on the inflammatory pathways. Moreover, *NLRC5* is found to be a transcription coactivator for the MHC class I gene. MHC class I receptor plays a key role cancer immune response [8, 9]. Under expression of *NLRC5* will cause impaired MHC class I activity, thus, increased risk for cancer and result in poor prognosis [9]. One study on breast cancer found that the promotion of *NLRC5* that is done by INF-*y* which in turn will upregulate the MHC class I receptors, thus increasing the effectiveness of cancer immunotherapy [10].

Salt inducible kinase 1 (*SIK1*) is a part of the AMP-activated protein kinase family (*AMPK*), which have been found to play a vital role in maintaining normal metabolic function and cellular growth [11]. Several studies have investigated the role of *SIK* in breast cancer; they found that a reduction in the expression of SIK is linked to metastatic disease and poor prognosis. While in the other hand, higher expression levels have a tumor suppressor effect [12]. *SIK1* has shown to stimulate the oxidative phosphorylation, which will result in the inhibition of breast cancer cell proliferation via inhibiting the glycolysis. Moreover, *SIK1* has direct interaction with P53 that results in positive regulation of the transcriptional activity of P53 that causes oxidative phosphorylation in the breast cancer cells. On the other hand, knockdown of P53 and *SIK1* will cause increased proliferation of cancer cells. However, the interaction of *SIK1* with mTOR signaling showed increased glycolysis and enhanced cell proliferation. These finding suggests the vital role of SIK in the regulation of glycolysis and cells proliferation [11].

Family-based studies have been the primary focus of study in the search for genetic determinants in breast cancer, but with new technologies that enable analysis of hundreds of thousands of SNPs, together with insights into the structure of genomic variation in the human genome, it is now possible to scan across the genome in search of common genetic variants associated with disease risk [13]. In this context, it was reported that hereditary breast and ovarian cancer syndromes can be caused by loss-of-function germline mutations in one of two tumor-suppressor genes, *BRCA1*, and *BRCA2* [14]. Besides, inherited mutations in *BRCA1* or *BRCA2* predispose to breast, ovarian, and other cancers. That is because *BRCA1* or *BRCA2* expressed protein products are implicated in processes fundamental to all cells, including DNA repair and recombination, checkpoint control of cell cycle, and transcription [15].

Cerda-Floris et al. reported that SNP, rs1501299 was associated with a risk of developing breast cancer in Mexican patient [16].

Liu et al. studied the SNP, rs799917, in BRCA1, and found this polymorphism to be associated and increased susceptibility to lung cancer in a Han Chinese population in the Liaoning Province of China [17].

There is lack of enough studies that investigate the possible association of significant SNPs with development of breast cancer in Arab patients. Therefore, we did this study to investigate the possible associated SNPs with development of breast cancer in our population of patients at the eastern region of Saudi Arabia. Although our patients' numbers were limited, our results led to finding suggested candidate biomarkers for possible prediction of breast cancer among Arab patients in our geographical region.

## 2. Materials and Methods

Study patients' sample were 26 Saudi females, ranged in age from 32 to 77 years old, with histologically confirmed newly diagnosed breast cancer. All patients (cases) were diagnosed at King Fahd Hospital of the University (KFHU), Khobar, KSA between January 2018 to December 2019.

The normal healthy controls (26 control volunteers) were age- and sex-matched with breast cancer cases. Healthy controls were assessed by a physician collaborator to make sure they are clinically healthy and not suspected to have any type of malignancy. Both cases and controls were asked through interview on a standardized questionnaire inquiring on their risk factors (diet, alcohol and tobacco use, medical history, family history of cancer, reproductive health, occupation, and environmental factors).

Paraffin-embedded tissue sections were obtained from the pathological masses of breast cancer cases for molecular studies. On the other hand, 5 mL peripheral blood samples collected from the healthy control volunteers were immediately stored at -80°C until molecular analysis. Clinical data of the cases (age, histopathological diagnosis, immunohistochemistry for estrogen receptor, progesterone receptors, and HER2/neu) were retrieved from clinical records and histopathology reports. Ethical clearance was obtained from the Institutional Review Board (No. IRB-2017-135-IRMC) of KFHU, and all participants gave written informed consents.

### 2.1. DNA Extraction and Genotyping Analysis

Genomic DNA from the blood samples were extracted and used for genotyping microarray for analyzing 243,345 exonic markers using human exome bead chip kit (v1.0 and v1.1, Illumina, San Diego, USA). All DNA samples were hybridized on the exome bead chip according to manufacturer's protocol. The hybridized samples on the exome chip were scanned using iScan (Illumina San Diego, USA). The data from the human exome bead chip was obtained using the iScan control software (Illumina, San Diego, USA). Instruments at the genetic research laboratory of the Institute for Research and Medical Consultations (IRMC), Imam Abdulrahman Bin Faisal University, was used for the DNA isolation, microarray genotyping, and analysis as described earlier [18, 19]. GenomeStudio 2.0 Data Analysis Software (Illumina, USA) was used for the initial quality verification of the call rate. Due to a call rate of 0.99 percent, 2 patients with breast cancer were eliminated from the study. With a 1 degree of freedom genotypic chi-squared test, the Hardy-Weinberg equilibrium (HWE) was investigated individually in the case and control groups. SNP-Nexus [20, 21] was used to ensure that variations reported at a base pair location on the corresponding chromosome were reported in accordance with Genome (GRCh37.p13.) Reference Consortium Human Build 37. Using Haploview version 4.2 [22] and gPLINK version 2.050 [23], case-control association analyses were performed to assess the influence of various alleles and haplotypes. To keep the type I error rate, Bonferroni corrections or false discovery rate corrections were used to validate the *p* values of 243345 SNPs (adjusted = 0.05/243345 = 2.0510^−07^). Significant was defined as p values less than 2.0510^−07^.

## 3. Results

A total of 52 samples (26 histologically confirmed breast cancer cases matched with 26 clinically healthy controls) were included in this study. As shown in Table 1, the cases' ages ranged from 32 to 77 years old. The cases histological diagnoses, and their estrogen receptors, progesterone receptors, and HER2/neu expressions are indicated in Table 1.

**Figure 1:**
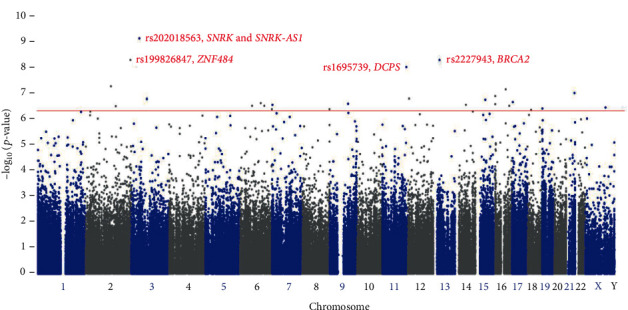
Manhattan plot of exonic 243,345 variants from association study with breast cancer in Saudi Arabians. Association is plotted according to position of the variant on each chromosome with −log10 (*p* values). The horizontal red line indicates the suggestive threshold. Colored SNP in red color denotes the most significant SNP with *p* ≤ 9.14 × 10^−09^.

Four SNPs [Chromosome 3: *SNRK* and *SNRK-AS1-*rs202018563G (*p*value = 6.97 × 10^−10^); Chromosome 13: *BRCA2-*rs2227943C (*p*value = 4.89 × 10^−09^); Chromosome 13: *ZNF484-*rs199826847C (*p*value = 4.91 × 10^−09^); and Chromosome 11: *DCPS-*rs1695739G (*p*value = 9.14 × 10^−09^)] were found to be highly associated significantly (*p*value ≤ 9.14 × 10^−09^) in patients with breast cancer even after Bonferroni corrections or false discovery rate corrections (corrected *α* = 0.05/243345 = 2.05 × 10^−07^) among the exonic variants 24,3345 studied (Figure 1; Table 2). All the associated SNPs obeyed the Hardy-Weinberg equilibrium. The most significant (*p*value ≤ 9.23 × 10^−07^) exonic variants that are associated in patients with breast cancer from the Saudi Arabians are listed in Table 2. Linkage disequilibrium analysis among SNPs with *p* ≤ 9.23 × 10^−07^ in Saudi Female with breast cancer revealed risk and protective haplotypes as listed in Table 3. The protective and risk haplotypes with 5 significant variants in the chromosome 2 and high degree (*p*value ≤ 7.50 × 10^−07^) of linkage disequilibrium includes: rs199826847A; rs189581518T; rs140626972A; rs115282281A; rs150343979C (Protective: *p* = 3.30 × 10^−08^), rs199826847G; rs189581518C; rs140626972C; rs115282281G; rs150343979T (risk: *p* = 7.50 × 10^−07^) (Table 3).

## 4. Discussion

Genetic heterogeneity in Arab populations on various disorders including cancers are common [24]. Hence, studying the genes and impact on diseases among them is challenging. The present study aimed to identify the genetic association on histologically confirmed breast cancer among the Saudi Arabians. The study has successfully identified candidate variants on breast cancer including variants in *BRCA2* gene. The mutation of *BRCA2* gene mutations account for around 20-40% of familial breast cancer cases. Moreover, the carriers of *BRCA2* mutations have a 45-49% risk to develop several types of cancer during their lifes [25]. Carriers of *BRCA2* mutation management include frequent screening, prophylactic surgeries in some cases, and genetic testing and counseling for other family members. There are numerous variants that are inferred from the sequencing data alone. Thus, those variants are called variants of uncertain significance (VUS) [25]. In parallel with our study, one group has investigated the prevalence of *BRCA* gene mutation in Saudi women with breast cancer; they found that mutation of *BRCA2* gene was found in 7 patients out of 310 with total percentage of both *BRCA1* and *BRCA2* of 12.9%; the percentage of *BRCA2* was 2.2%. This result is correlated with same percentage that found in Lebanese population but found to be higher than the Qatari population [26]. Another study that has been conducted on Gulf region population has investigated the prevalence of *BRCA* mutations in women with ovarian cancer; the result showed that the 15 out of 88 women had *BRCA* mutation with the total percentage of 17%; *BRCA* was accounted for 9.1%; this result showed higher than those reported in global studies [27].

Our current study revealed the most significant *SNP*, rs202018563 in the gene, *SNRK*, is the sucrose nonfermenting 1-related kinase. *SNRK* is considered as protein kinase that has significant role in signal transduction through the phosphorylation of certain amino acid and protein phosphorylation. *SNRK* plays vital role in the regulation of different cellular processes such as cellular proliferation, differentiation, and metabolism. *SNRK* is a member of the AMP-activated protein kinase family. Historically, the first identification of *SNRK* was in 1966 where it was discovered in adipocyte, and its expression played a role in the differentiation of cells into adipose-like cells [28].

It is also suggested that *SNRK* regulates the transportation of glucose and cell motility. Of note, the expression of *SNRK* is found to be associated with cancer disease and obesity [29]. In addition to our findings, some investigators have reported that *SNRK* has been found to be expressed in ovarian cancer cell lines [28, 29]. The proposed explanation is that *SNRK* is regulated by liver kinase B1 (LKB1) which function is to suppress the signaling pathway. One study has found that mutated *LKB1* could alter several kinases pathways including SNRK, and it is associated with breast cancer in which it can affect the patient survival and the outcome of the treatment [30].

Our results showed that SNP, rs1695739 in *DCPS* is one of the significant variants in our breast cancer patients. *DCPS* is the decapping enzyme that is part in the mRNA decay process, which is the process that is responsible for the degradation of the mRNA in mammalian cells. *DCPS* is responsible for the decapping of the cap structure that is generated by 3′ to 5′ exonucleolytic degradation [31]. Any change in the rate of mRNA degradation process can alter the expression level of different pathways which in turn affect the cellular function [31]. Interestingly, mutation in the *DCPS* gene has been reported with neurological malfunction and affecting normal recognition processes, and it is implicated in the spinal muscular atrophy disease [32]. Moreover, one study showed that the *DCPS* activity is essential for AML cell survival. Therefore, it was suggested that targeting *DCPS* could serve as treatment for AML [33].

Concerning our reported breast cancer-associated variant SNP, rs189581518 on *ZRANB3* gene. *ZRANB3* belongs to the family of sucrose nonfermenting 2 group of ATPase and is considered as nuclease that has role in DNA replication and DNA repair [34]. *ZRANB3* interacts with proliferating cell nuclear antigen (PCNA) which is a processivity factor for DNA polymerase. PCNA has a role in controlling the cellular response during replication in the case of DNA damage. *ZRANB3* recruited to interact with PCNA in sites where there are DNA breaks and stress on the replication fork. *ZRANB3* malfunction results in a DNA that is sensitive to being damaged by DNA damaging agents [34]. *ZRANB3* variants have been found to be associated with several types of cancers such as endometrial carcinoma [34]. These findings support our observation about the breast cancer-associated significant SNP, rs189581518 on *ZRANB3* gene. The analysis of *ZRANB3* variants through the bioinformatics approach has suggested that these variants are associated with pathogenicity most of the time [35]. One study has investigated the association of *BRCA2* gene mutation and the deficit in the DNA repriming where *ZRANB3* and other repairing factors are depleted; they found that these cells are having increased risk of DNA instability in the form of chromatid breaks (CTB) after radiation in patient with breast cancer suggesting the association of this defect to play role in the tumor suppression and response to treatment [36]. Another study which supports our findings showed that the *BRCA1* or *BRCA2* deficit cells and depletion of SNF2 family fork remodelers which includes *ZRANB3* could increase the DNA degradation and might explain the insights of genomic instability that found in *BRCA1* and BRCA*2* mutated cells [37].

*DMXL2* is a newly discovered regulator of the notch signaling pathway. The notch signaling has been reported to be disrupted frequently in breast cancer, that is estrogen receptor positive [38]. Moreover, it is implicated for therapy resistance, which is a challenging issue in the treatment of breast cancer. There are enormous efforts to target this pathway to improve the prognosis and outcome of breast cancer. Studies have shown that *DMXL2* is highly expressed in resistant breast cancer, and *DMXL2* enhances the transition of the epithelium into mesenchymal via the activation of notch signaling. It has been reported that reduction in the expression of *DMXL2* will decrease the notch signaling significantly, thus, improving the outcome of breast cancer treatment [38]. The significant SNPs, rs114516513 in *DMXL2* was observed in the study, and previous expression studies indicate the need of further studies in the Arab ancestries with breast cancer. The glycogen branching enzyme (*GBE1*) is thought to be a major regulator of cancer microenvironment; the tumor microenvironment is a complex of cells and factors that enables tumor growth and development [39]. Inside the microenvironment, tumor cells will restrict the activity of T cells through different metabolic pathways adaptations; one important metabolic pathway is the glycogen metabolism [40]. *GBE1* knockdown is shown to be correlated with increased and enhanced immune response, thus inhibiting, and limiting the growth of the cancer cells [39]. The mutations in *GBE1* have been reported with several types of cancers including lung adenocarcinoma [39, 40] and melanoma [41].

Even though, although our samples' size is limited, our findings of significant SNPs among patients with breast cancer even after Bonferroni corrections suggest the importance of further detailed larger samples analysis for significant SNPs in the Arab ancestries with breast cancer.

## 5. Conclusion

Our exome wide biomarkers study identified 4 SNPs [*SNRK* and *SNRK-AS1-*rs202018563G; *BRCA2-*rs2227943C; *ZNF484-*rs199826847C; and *DCPS-*rs1695739G] as the most significant SNPs among our patients with breast cancer compared to the healthy controls. Although our patients' numbers were limited, the identified SNPs might shed some light on certain breast cancer-associated functional multigenic variations in Arab patients. These associated SNPs in Arab breast cancer patients were found even after Bonferroni corrections, indicating the need for more extensive large-scale investigation of significant SNPs to reveal the candidate biomarkers for the prediction of breast cancer among Arab individuals.

## Figures and Tables

**Table 1:** Histopathological and immunohistochemistry characteristics of breast cancer cases. Abbreviations: IDC: invasive ductal carcinoma; NOS: not otherwise specified; ILC: invasive lobular carcinoma; HER2/neu: human epidermal growth factor receptor 2.

Age (year-old)	Diagnosis	Estrogen receptors	Progesterone receptors	HER2/neu
55	IDC, NOS	Pos. 100%	Pos. 85%	Neg.
77	ILC, with pleomorphic features	Pos. >90%	Neg.	Neg.
60	IDC, NOS	Pos. 1%	Neg.	Neg.
68	IDC, NOS	Pos. 90%	Neg.	Neg.
53	IDC, NOS, with focal mucinous changes	Pos. 60%	Pos. 10%	Neg.
58	IDC, NOS	Pos. 70%	Pos. 65%	Neg.
51	IDC, NOS	Neg.	Neg.	Pos.
60	IDC, NOS	Pos. 100%	Pos. 80%	Neg.
33	IDC, NOS	Neg.	Neg.	Pos.
53	IDC, micropapillary type	Neg.	Neg.	Neg.
54	Invasive solid papillary carcinoma, with mucinous component	Pos. 95%	Pos. 95%	Neg.
66	IDC, NOS	Pos. 90%	Pos. 1%	Neg.
48	IDC, NOS	Pos. 90%	Pos. 90%	Neg.
45	IDC, NOS	Pos. 95%	Pos. 10%	Neg.
32	IDC, NOS	Pos.	Pos.	Neg.
56	IDC, with medullary features	Pos. 85%	Pos. 10%	Pos.
53	IDC, NOS	Pos. 85%	Neg.	Neg.
52	IDC, NOS	Pos. 90%	Pos. 40%	Neg.
39	Medullary carcinoma	Neg.	Neg.	Neg.
55	Mucinous carcinoma	Pos. 95%	Pos. 80%	Pos.
55	IDC, NOS	Pos. 80%	Pos. 80%	Pos.
38	Invasive carcinoma with neuroendocrine features	Pos. 90%	Pos 90%	Neg.
45	IDC, NOS	Neg.	Neg.	Neg.
63	IDC, NOS	Pos. 90%	Pos. 90%	Neg.
71	IDC, NOS	Pos. 90%	Pos. 5%	Neg.
64	IDC, NOS	Pos. 90%	Pos. 70%	Pos.

**Table 2:** The most significant SNPs associated with breast cancer in Saudi Arabians.

S. no	CHR	SNP ID	BP	MA	MAF	*p* value	OR(L95-U95)	Gene	AA	CCF
1	3	rs202018563	43389699	A	0.482	6.97E-10	18.9 (6.49-55.01)	*SNRK* and *SNRK-AS1*	G	0.875, 0.270
2	13	rs2227943	32911278	T	0.415	4.89E-09	0.05 (0.01-0.17)	*BRCA2*	C	0.806, 0.214
3	2	rs199826847	239049921	T	0.473	4.91E-09	10.17 (3.95-26.11)	*ZNF484*	C	0.868, 0.347
4	11	rs1695739	126196175	A	0.383	9.14E-09	25.67 (6.74-97.79)	*DCPS*	G	0.875, 0.214
5	2	rs189581518	136111018	A	0.394	5.17E-08	11.07 (4.09-29.92)	*ZRANB3*	C	0.808, 0.235
6	16	rs7206703	57091977	T	0.4	6.83E-08	0.04 (0.01-0.19)	*NLRC5*	C	0.947, 0.439
7	21	rs112011493	44840296	C	0.474	9.63E-08	0.09 (0.03-0.24)	*SIK1*	T	0.841, 0.329
8	16	rs762349227	682670	T	0.382	1.24E-07	0.04 (0.01-0.20)	*WFIKKN1*	C	0.767, 0.222
9	12	rs200582033	6031943	C	0.311	1.56E-07	18.91 (5.20-68.72)	*ANO2*	T	0.978, 0.535
10	15	rs114516513	51758447	T	0.397	1.61E-07	0.05 (0.01-0.22)	*DMXL2*	C	0.889, 0.220
11	3	rs192044702	81698005	A	0.185	1.70E-07	12.43 (4.41-35.01)	*GBE1*	G	0.542, 0.059
12	17	rs139171143	4802111	C	0.3	2.10E-07	19.69 (5.38-72.1)	*CHRNE* and *C17orf107*	G	0.600, 0.129
13	6	rs140709825	111697900	A	0.357	2.28E-07	12.2 (4.35-34.24)	*REV3L*	G	0.719, 0.182
14	9	rs142712699	95610753	A	0.256	2.46E-07	14.17 (4.39-45.65)	*ZNF484*	C	0.722, 0.117
15	16	rs11552432	1823054	C	0.186	2.53E-07	44 (7.28-266.1)	*MRPS34, EME2* and *NME3*	G	0.800, 0.083
16	7	rs141963459	2691854	G	0.02	2.64E-07	21.64 (4.56-102.7)	*TTYH3*	A	1.000, 0.972
17	14	rs35064097	57700585	T	0.207	2.71E-07	10.81 (4.06-28.79)	*EXOC5*	G	0.455, 0.056
18	6	rs200026839	66204932	T	0.385	2.94E-07	11.5 (4.18-31.64)	*EYS*	G	0.767, 0.212
19	16	rs16957552	75269124	T	0.365	2.94E-07	0.05 (0.01-0.22)	*BCAR1*	C	0.950, 0.488
20	6	rs57738384	129763368	A	0.286	2.96E-07	10.13 (3.73-27.52)	*LAMA2*	G	0.700, 0.156
21	2	rs140626972	160602359	A	0.211	3.03E-07	20 (5.68-70.42)	*7-mar*	G	0.577, 0.062
22	23	rs145970300	107819173	A	0.151	3.43E-07	10.36 (3.81-28.17)	*COL4A5*	C	0.389, 0.029
23	19	rs114544630	1481787	G	0.429	3.72E-07	11.67 (4.08-33.29)	*PCSK4*	C	0.900, 0.419
24	8	rs187011732	144992465	C	0.449	3.99E-07	0.08 (0.02-0.24)	*PLEC*	T	0.875, 0.385
25	6	rs562092150	170115902	A	0.118	4.03E-07	39.5 (4.88-319.5)	*PHF10*	C	0.367, 0.014
26	18	rs201319761	19997762	A	0.145	4.28E-07	42 (6.55-269.3)	*CTAGE1*	G	0.406, 0.038
27	14	rs146398509	92470845	T	0.282	4.85E-07	12.71 (4.15-38.87)	*TRIP11*	C	0.579, 0.125
28	2	rs115282281	26534041	T	0.219	4.89E-07	0.06 (0.02-0.23)	*ADGRF3* and *LOC105374334*	C	0.500, 0.079
29	1	rs202005618	226411686	G	0.349	5.13E-07	9.62 (3.72-24.89)	*MIXL1*	C	0.952, 0.500
30	9	rs56170708	96010036	G	0.351	5.51E-07	0.07 (0.02-0.24)	*WNK2*	A	0.935, 0.500
31	7	rs41273999	23821123	A	0.351	5.68E-07	0.025 (0.003-0.19)	*STK31*	G	0.769, 0.191
32	15	rs11574476	73994778	G	0.337	6.01E-07	17.11 (4.42-66.15)	*CD276*	A	0.731, 0.194
33	12	rs7302017	63004583	A	0.214	6.34E-07	10.96 (3.96-30.32)	None	G	0.800, 0.087
34	15	rs55799438	40544493	A	0.415	6.58E-07	11.48 (3.99-33.03)	*C15orf56*, *PAK6* and *BUB1B-PAK6*	G	0.938, 0.432
35	18	rs138472116	9124917	T	0.196	6.72E-07	0.08 (0.03-0.25)	*NDUFV2* and *NDUFV2-AS1*	C	0.472, 0.066
36	2	rs150343979	25384086	A	0.45	6.95E-07	15.19 (5.56-41.44)	*POMC*	G	0.909, 0.342
37	4	rs75428449	175224971	A	0.337	7.05E-07	28.36 (5.64-142.6)	*CEP44*	C	0.731, 0.194
38	5	rs199715117	130517944	A	0.255	7.29E-07	11.24 (3.96-31.97)	*LYRM7*	C	0.559, 0.111
39	7	rs34850251	94164820	A	0.337	7.87E-07	12.42 (4.16-37.12)	*CASD1* and *LOC105375404*	C	0.731, 0.182
40	5	rs147680491	61779069	A	0.29	7.89E-07	0.10 (0.03-0.27)	*IPO11*	G	0.633, 0.143
41	23	rs146662506	11207098	A	0.365	9.23E-07	14.93 (4.71-47.38)	*ARHGAP6*	G	0.719, 0.188

CHR: Chromosome; SNP ID: Single nucleotide polymorphism ID; BP: Base pair position at the respective chromosome as per GRCh37.p13; MA: Minor allele name; MAF: Frequency of minor allele in controls; OR: Odd ratio; SE: Standard error; L95: Lower bound of 95% confidence interval for odds ratio; U95: Upper bound of 95% confidence interval for odds ratio. AA: Associated Allele; CCF: Case, Control Frequencies.

**Table 3:** Haplotypes of SNPS with the significance *p* ≤ 9.23 × 10^−07^ in Saudi females with breast cancer.

CHR	Block	Haplotype	Case, control frequencies	Chi Square	*p* value	Haplotypes	Risk/protective
2	Block 1	ATAAC	0.068, 0.577	30.524	3.30E-08	rs199826847A; rs189581518T; rs140626972A; rs115282281A; rs150343979C^∗^	Protective
		GCAAT	0.271, 0.201	0.796	0.3724	rs199826847G; rs189581518C; rs140626972A; rs115282281A; rs150343979T	
		GCCGT	0.365, 0.031	24.483	7.50E-07	rs199826847G; rs189581518C; rs140626972C; rs115282281G; rs150343979T^∗∗^	Risk
		GCAGT	0.074, 0.020	2.245	0.134	rs199826847G; rs189581518C; rs140626972A; rs115282281G; rs150343979T	
		ATAAT	0.045, 0.033	0.111	0.7396	rs199826847A; rs189581518T; rs140626972A; rs115282281A; rs150343979T	
		GCCAT	0.077, 0.010	3.784	0.0518	rs199826847G; rs189581518C; rs140626972C; rs115282281A; rs150343979T	
		GTCAC	0.014, 0.044	0.785	0.3757	rs199826847G; rs189581518T; rs140626972C; rs115282281A; rs150343979C	
		GTAGT	0.043, 0.025	0.283	0.5945	rs199826847G; rs189581518T; rs140626972A; rs115282281G; rs150343979T	
		GTCAT	0.021, 0.028	0.058	0.8091	rs199826847G; rs189581518T; rs140626972C; rs115282281A; rs150343979T	
		GCCAC	0.005, 0.015	0.254	0.6144	rs199826847G; rs189581518C; rs140626972C; rs115282281A; rs150343979C	

3	Block 1	AA	0.139, 0.701	37.832	7.71E-10	rs202018563A; rs192044702A	
		GG	0.543, 0.130	25.633	4.13E-07	rs202018563G; rs192044702G	
		GA	0.319, 0.169	3.88	0.0489	rs202018563G; rs192044702A	

5	Block 1	AA	0.327, 0.817	29.093	6.90E-08	rs199715117A; rs147680491A^∗^	Protective
		GC	0.562, 0.138	24.361	7.99E-07	rs199715117G; rs147680491C^∗∗^	Risk
		AC	0.100, 0.029	2.702	0.1002	rs199715117A; rs147680491C	
		GA	0.011, 0.015	0.032	0.8581	rs199715117G; rs147680491A	

6	Block 1	TAAA	0.161, 0.710	29.845	4.68E-08	rs140709825T; rs200026839A; rs57738384A; rs562092150A^∗^	Protective
		GGGA	0.325, 0.181	2.887	0.0893	rs140709825G; rs200026839G; rs57738384G; rs562092150A	
		GGGC	0.213, 0.006	16.031	6.23E-05	rs140709825G; rs200026839G; rs57738384G; rs562092150C^∗∗^	Risk
		TGGA	0.073, 0.044	0.429	0.5127	rs140709825T; rs200026839G; rs57738384G; rs562092150A	
		GAAA	0.060, 0.019	1.365	0.2426	rs140709825G; rs200026839A; rs57738384A; rs562092150A	
		TGGC	0.077, 0.000	6.078	0.0137	rs140709825T; rs200026839G; rs57738384G; rs562092150C	
		GGAC	0.046, 0.014	1.091	0.2963	rs140709825G; rs200026839G; rs57738384A; rs562092150C	
		GGAA	0.042, 0.013	0.915	0.3389	rs140709825G; rs200026839G; rs57738384A; rs562092150A	
		TGAA	0.004, 0.014	0.224	0.6359	rs140709825T; rs200026839G; rs57738384A; rs562092150A	

7	Block 1	CAA	0.136, 0.743	36.338	1.66E-09	rs141963459C; rs41273999A; rs34850251A	
		CGC	0.711, 0.215	25.734	3.92E-07	rs141963459C; rs41273999G; rs34850251C	
		CGA	0.078, 0.008	4.009	0.0453	rs141963459C; rs41273999G; rs34850251A	
		CAC	0.073, 0.007	3.811	0.0509	rs141963459C; rs41273999A; rs34850251C	
		TAA	0.002, 0.027	0.838	0.3599	rs141963459T; rs41273999A; rs34850251A	

9	Block 1	CA	0.665, 0.273	19.393	1.06E-05	rs142712699C; rs56170708A^∗∗^	Risk
		AG	0.065, 0.502	25.401	4.66E-07	rs142712699C; rs56170708G^∗^	Protection
		AA	0.269, 0.224	0.338	0.5611	rs142712699A; rs56170708A	

12	Block 1	TG	0.626, 0.299	13.751	2.00E-04	rs200582033T; rs7302017G	Risk
		CA	0.022, 0.442	26.444	2.71E-07	rs200582033C; rs7302017A^∗^	Protection
		TA	0.331, 0.235	1.458	0.2273	rs200582033T; rs7302017A	
		CG	0.021, 0.024	0.017	0.8952	rs200582033C; rs7302017G	

14	Block 1	TT	0.379, 0.848	29.01	7.20E-08	rs35064097T; rs146398509T^∗^	Protection
		GC	0.412, 0.061	23.087	1.55E-06	rs35064097G; rs146398509C^∗∗^	Risk
		TC	0.172, 0.075	2.761	0.0966	rs35064097T; rs146398509C	
		GT	0.037, 0.016	0.553	0.4571	rs35064097G; rs146398509T	

15	Block 1	GCA	0.733, 0.189	28.894	7.65E-08	rs114516513G; rs11574476C; rs55799438A^∗∗^	Risk
		ATG	0.063, 0.473	16.566	4.70E-05	rs114516513A; rs11574476T; rs55799438G^∗^	Protection
		GTA	0.146, 0.113	0.224	0.6359	rs114516513G; rs11574476T; rs55799438A	
		ATA	0.059, 0.130	1.18	0.2774	rs114516513A; rs11574476T; rs55799438A	
		GTG	0.000, 0.095	3.241	0.0718	rs114516513G; rs11574476T; rs55799438G	

16	Block 1	CCTT	0.050, 0.443	19.475	1.02E-05	rs7206703C; rs762349227C; rs11552432T; rs16957552T^∗^	Protection
		TGTC	0.419, 0.236	4.451	0.0349	rs7206703T; rs762349227G; rs11552432T; rs16957552C	
		TGCC	0.326, 0.057	16.473	4.93E-05	rs7206703T; rs762349227G; rs11552432C; rs16957552C^∗∗^	Risk
		TCTC	0.076, 0.121	0.601	0.4382	rs7206703T; rs762349227C; rs11552432T; rs16957552C	
		TCCC	0.127, 0.048	2.561	0.1096	rs7206703T; rs762349227C; rs11552432C; rs16957552C	
		CCCT	0.000, 0.057	2.365	0.1241	rs7206703C; rs762349227C; rs11552432C; rs16957552T	
		CCTC	0.002, 0.027	0.89	0.3456	rs7206703C; rs762349227C; rs11552432T; rs16957552C	

18	Block 1	TA	0.449, 0.889	28.425	9.74E-08	rs201319761T; rs138472116A^∗^	Protection
		TG	0.188, 0.070	3.971	0.0463	rs201319761T; rs138472116G	
		CG	0.272, 0.008	22.621	1.97E-06	rs201319761C; rs138472116G^∗∗^	Risk
		CA	0.091, 0.033	1.924	0.1654	rs201319761C; rs138472116A	

23	Block 1	AA	0.309, 0.791	29.009	7.21E-08	rs145970300A; rs146662506A^∗^	Protection
		AG	0.319, 0.130	6.65	0.0099	rs145970300A; rs146662506G	Risk
		CG	0.373, 0.079	17.124	3.50E-05	rs145970300C; rs146662506G^∗∗^	Risk

CHR: Chromosome number. ^∗∗^Risk haplotypes (*p* < 1.0 × 10 − 4) and ^∗^protective haplotypes (*p* < 1.0 × 10^−4^) with opposite alleles.

## Data Availability

All data are available on request through the corresponding author.
